# Efficient Bioimaging with Diaminodicyanoquinodimethanes: Selective Imaging of Epidermal and Stomatal Cells and Insight into the Molecular Level Interactions

**DOI:** 10.1038/s41598-017-11293-y

**Published:** 2017-09-06

**Authors:** N. Senthilnathan, Ch. G. Chandaluri, T. P. Radhakrishnan

**Affiliations:** School of Chemistry, University of Hyderabad, Hyderabad, 500 046 India

## Abstract

The enhanced fluorescence emission of diaminodicyanoquinodimethanes (DADQs) in rigid and aggregated states holds great promise for bioimaging applications. This is demonstrated through their efficient application in epidermal and stomatal imaging with selective staining of cell walls and nuclei. Major advantages include the small quantities (a few nmols) of the fluorophore required, choice of DADQs soluble in water and organic solvents, and quick staining of the specimen in buffer-free state and in buffer medium. The molecular level interactions that enable staining are unraveled through isothermal calorimetry, infra-red spectroscopy and microscopy with energy dispersive X-ray spectroscopy analysis. It is proposed that DADQs with ionic or H-bonding functionalities bind to the polygalacturonic acid moieties in the epidermal layer; the former can bind also to nucleic acid polyanions. Fluorescence experiments explain the emission enhancement that enables the efficient imaging. DADQs are easy to synthesize, non-cytotoxic, and thermally, chemically and photo-stable, requiring no special storage conditions; preliminary experiments point to their potential utility in imaging different classes of cells.

## Introduction

Fluorescence based imaging is a prominent tool for the investigation of the structure and function of biological systems^1^. Issues related to cytotoxicity, photo-stability and emission quenching have limited the number of fluorophores that can be deployed in practical and efficient imaging applications. Fluorescent protein based probes are mostly expensive, and often suffer from low molar absorptivity, instability during sample fixation that may involve denaturants, potential interference with cell functions, and undesirable sensitivity to factors like temperature and pH^2–5^. Even though quantum dots are highly photo-stable and emissive, they are generally plagued by toxicity issues^6^; nanoparticles based on small organic molecules and macromolecules are emerging as viable alternatives^7^. Small molecule based fluorophores are relatively easy to synthesize and characterize, and afford the flexibility to incorporate desired functionalities and interactions with the biological systems; however most are susceptible to aggregation-induced self-quenching of fluorescence emission. The limited classes of molecules that exhibit strong fluorescence in the aggregated/solid states (often called aggregation-induced emission enhancement) include tetraphenylethenes^8^, diphenylbutadienes^9^, hexaphenylsilole^10^ and diaminodicyanoquinodimethanes (DADQs). We have reported on the strong fluorescence of DADQs in crystals^11^, nanocrystals^12^, amorphous particles^13^ and thin films^14, 15^; the critical role of specifically oriented aggregation in the fluorescence enhancement has been demonstrated recently^16^. DADQs are potential candidates for efficient bioimaging.

An important and illustrative case that we have considered is the imaging of stomata in dicotyledon plant leaves; the stomatal apparatus and epidermal cells, as well as organelles like mitochondria and nuclei are relevant targets. Stomatal imaging is critical for morphological and epidermal studies of plant species, understanding the stimuli responsive dynamics of inner/outer guard cell walls and related signal transduction pathways^17^, and stomatal development issues like deposition pattern of callose in the guard cell wall^18^. Small molecule based fluorophores such as propidium iodide, safranin, aniline blue, DAF-2DA, BCECF-AM, H2DCF-DA, calcoflour white and acridine orange have been popular choices for imaging epidermal constituents^19–24^. Shortcomings of many of these probes include high cost and specialized storage conditions like low temperature and protection from light^25, 26^, the need to use non-aqueous, toxic solvents like DMSO^27^, carcinogenicity and mutagenicity^28^; aggregation-induced quenching of fluorescence due to factors such as self-absorption, excimer/exciplex formation and energy transfer is a problem in most cases. In addition to low cost and easy storage, the critical attributes of an ideal dye for fluorescence imaging of stomata include hydrophilicity to avoid binding to membranes, functionalities like ionic groups to selectively interact or bind with the cell wall, nucleus etc. and aqueous solubility to enable simple staining protocols.

**Figure 1:**
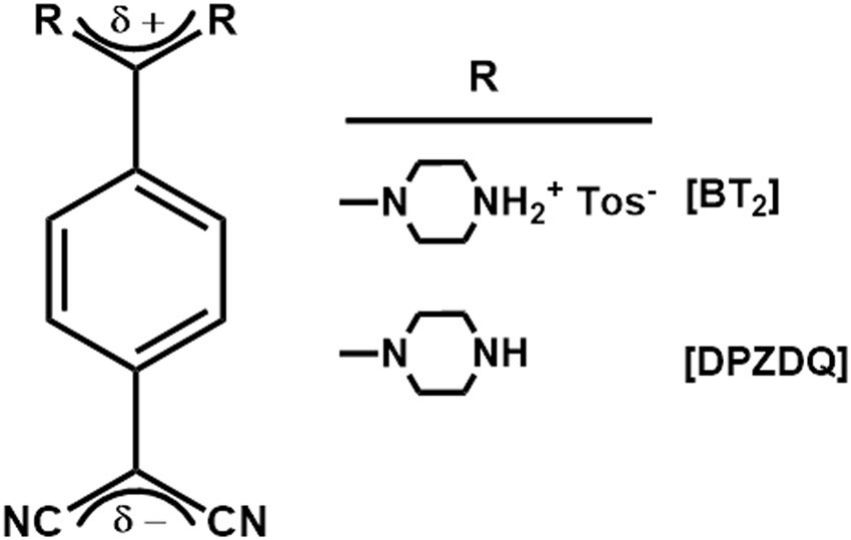
Molecular structures. Molecular structure of the DADQs, BT_2_ and DPZDQ.

**Figure 7:**
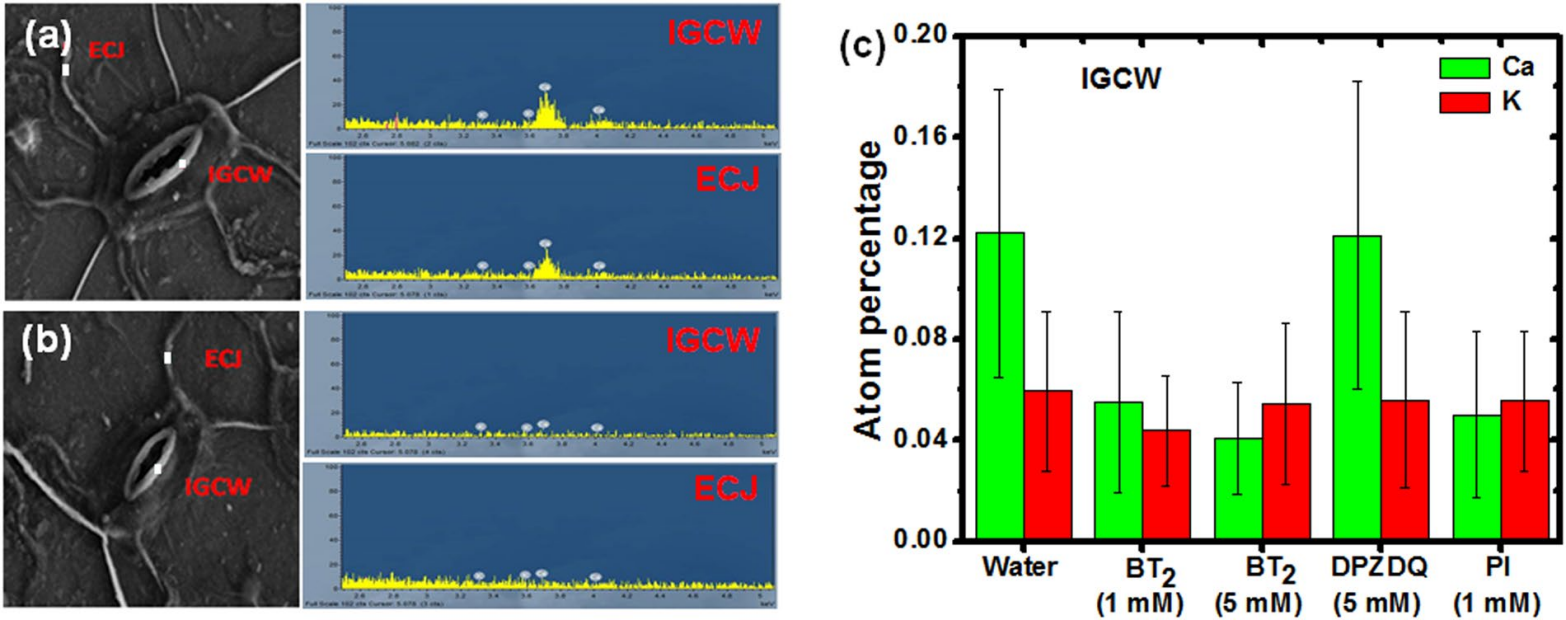
Microscopy and analysis. FESEM image and EDX spectra of selected regions (ECJ: epidermal cell junction; IGCW: inner guard cell wall) shown in the image, of pea epidermis (**a**) placed in pure water, and (**b**) stained using BT_2_ solution in water (0.5 mL of 5 mM) for 2 min. (**c**) Average Ca and K content in the IGCW region of the pea epidermis under different conditions.

DADQs are very relevant in this context. First reported in the 1960s^29^, they can be directly synthesized from commonly available precursors; the simple molecular structure and design flexibility including hydrophilic/water soluble derivatives^11^ imply low cost of preparation and usage. The high thermal and photochemical stability are important from an application point of view. The enhancement of fluorescence from solution to aggregated/solid states is uniquely relevant for imaging, as high concentrations of the dye can be used without self-quenching problems; even though a few aggregation-induced emission based luminogens have been developed as labels for organelles like membrane and mitochondria^30, 31^, HeLa cells and MCF-7 breast cancer cells^32^, and extracellular calcium ions^33^, there are no examples of stomatal imaging. We demonstrate high contrast, spatially well-resolved and selective imaging of stomatal and epidermal cell walls and nuclei in the epidermal layer of pea (*Pisum sativum* L. cv. Arkel) leaf, using the DADQ derivative, 7,7-bis(piperazinium)-8,8-dicyanoquinodimethane bis(*p*-toluene sulfonate) (B^2+^[Tos^−^]_2_ or simply BT_2_, Fig. 1)^34^. General utility of DADQs is illustrated using the other derivative shown in Fig. 1, 7,7-bis(piperazine)-8,8-dicyanoquinodimethane (DPZDQ)^35^. Choice of the DADQ derivatives, BT_2_ and DPZDQ allow an unambiguous illustration of the critical relevance of strong electrostatic and H-bonding interactions that could lead to strong binding with biological molecules; the consequent rigid environment and aggregation effects would induce enhanced fluorescence emission in DADQs and facilitate effective imaging. The relatively low cost of the materials, small concentrations required for staining, solubility in aqueous and organic media, feasibility of quick and easy sample preparation in water or buffer media, and the high quality and selectivity of stomatal imaging achieved are highlighted; selective staining of specific cell structures by the choice of the medium as well as the solvent used to prepare the dye solution are significant. Calorimetry, microscopy and spectroscopy investigations, as well as control experiments with a range of DADQs, provide insight into the molecular level interactions that lead to the efficient and selective imaging. It is specifically demonstrated that binding with polygalacturonic acid and its salt are critical. Demethyl esterified pectin (calcium salt of polygalacturonic acid), the major component of the cell wall plays a dominant role in its dynamics; the calcium ions enhance the strength to handle the turgor pressure. Even though replacement of the calcium by ionic dye molecules during the staining can impair the cell viability, the concentrations of the DADQs that we employ do not affect the cells adversely. Finally, we establish the relevance of DADQ based imaging in the context of the autofluorescence response of pea leaf epidermal cells, the general applicability of DADQs in fluorescence imaging of various kinds of cells, and the stability and non-cytotoxic nature of these novel imaging dyes which are of great practical importance.

## Results and Discussion

**Figure 2:**
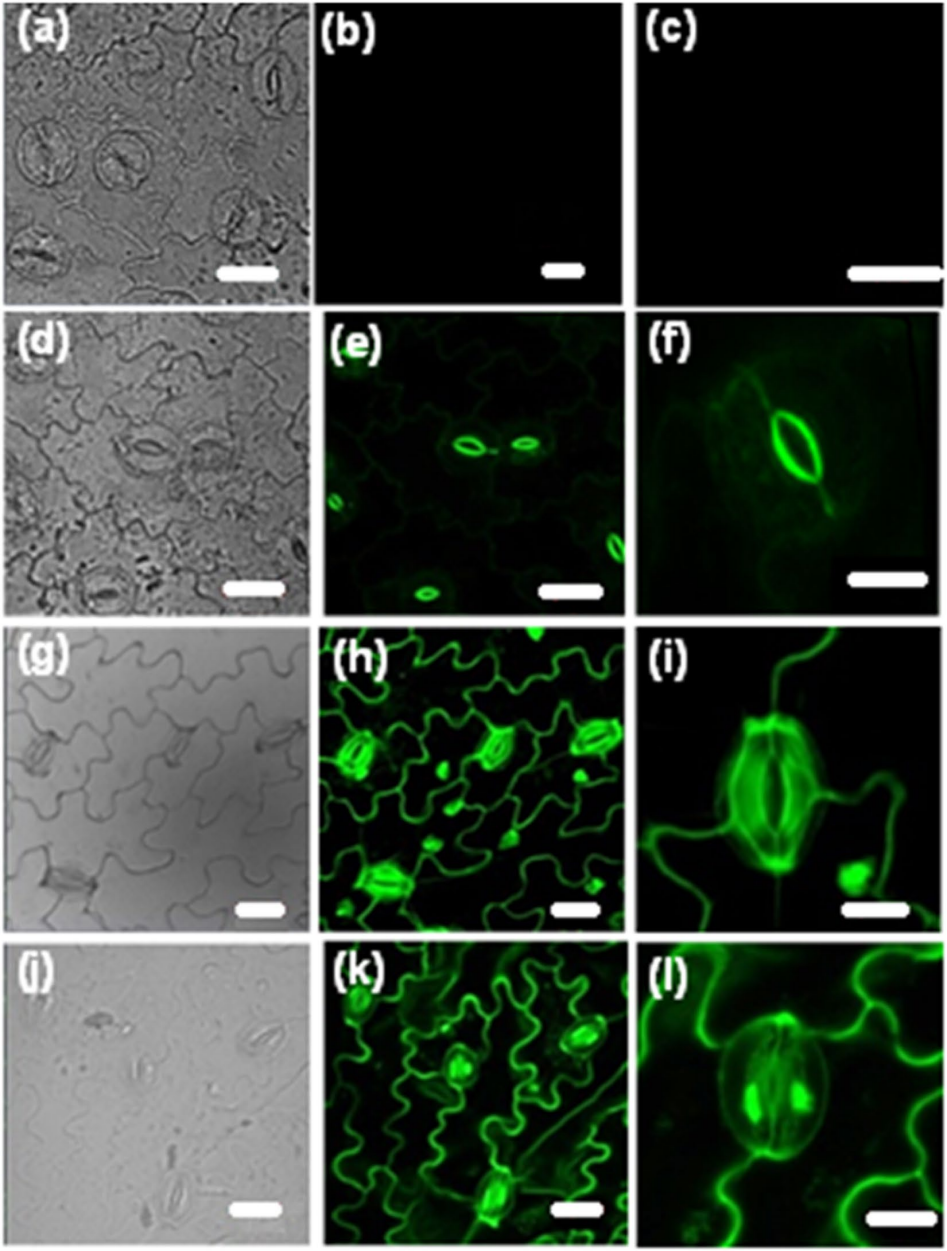
CLSM images. Images of pea epidermis in buffer-free state: (**a**–**c**) control, and stained using, (**d**–**f**) BT_2_ solution in water (15 µL of 0.05 mM), (**g**–**i**) BT_2_ solution in water (15 µL of 1 mM), and (**j**–**l**) BT_2_ solution in DMSO (15 µL of 1 mM); bright field images are shown in (**a**,**d**,**g**,**j**), and the fluorescence images at two magnifications in the remaining panels (excitation wavelength: 488 nm). (Scale bar: 10 µm in (**c**),(**f**),(**i**),(**l**); 20 µm in all others).

**Figure 3:**
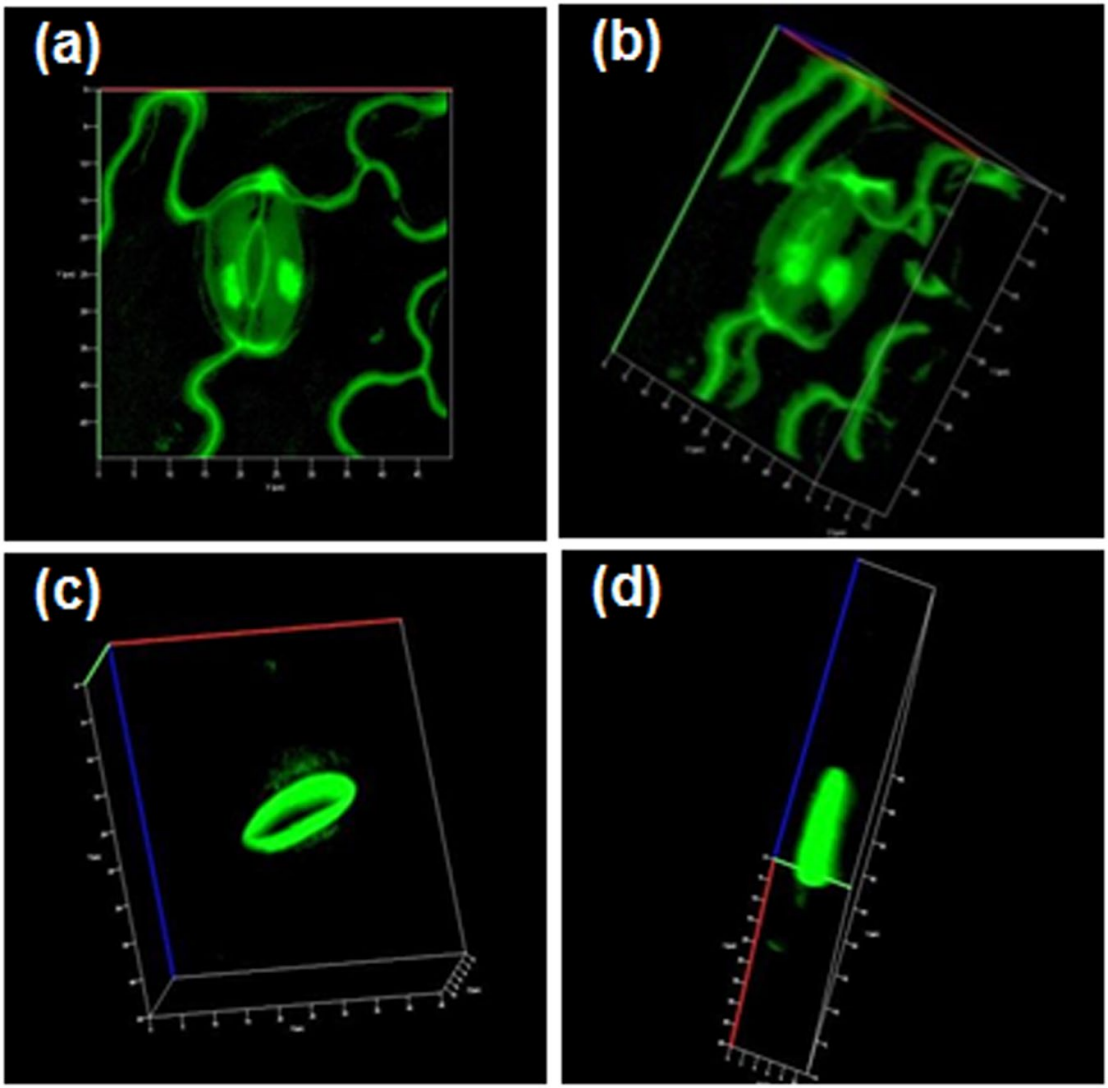
3-D CLSM images. 3-D reconstructions (at different viewing angles) from CLSM Z-stack images of the pea epidermis stained using (**a**,**b**) DMSO solution of BT_2_ (15 µL of 1 mM), in buffer-free state, and (**c**,**d**) aqueous solution of BT_2_ (15 µL of 1 mM) in buffer medium (excitation wavelength: 488 nm).

Imaging experiments with no special precaution taken to maintain the specimen alive by keeping in a buffer, are still useful for various epidermal and stomatal studies such as stomatal indexing and guard cell size and aperture measurements. This has the advantage that undesirable interactions between the buffer medium and the cell components, if any, are avoided. Therefore we have first tested the utility of the DADQs in imaging, by direct treatment of the prepared epidermal layer (see Methods section) in a buffer-free state, with the fluorophore solution. Very small quantities of BT_2_ as aqueous solution (15 µL of 0.05 or 1 mM) or as DMSO solution (15 µL of 1 mM) were spread directly on the epidermal layer of pea leaf placed on a microscope slide, kept for ~ 2 min, washed briefly to remove the excess dye, protected with a cover slip and imaged directly in a confocal laser scanning microscope (CLSM); processes like incubation for extended time are not required, and the amount of dye used is 0.75–15 nmol. Well resolved fluorescence images could be recorded (Fig. 2; bright field images are also shown). When the lower concentration of BT_2_ in water is used, only the inner wall of the guard cells is stained (Fig. 2d–f); with the higher concentration, walls of the guard cell, as well as walls and nuclei of the epidermal cells are stained clearly (Fig. 2g–i). BT_2_ solution in DMSO on the other hand, enters the guard cell and stains its nucleus as well (Fig. 2j–l); the 3-D view constructed using Z-stack images (Fig. 3a,b) shows clearly the staining of the nucleus. Interestingly, the staining by BT_2_ is found to persist in samples which were washed rigorously and repeatedly, showing clearly its strong binding, a point explored in detail later.

**Figure 4:**
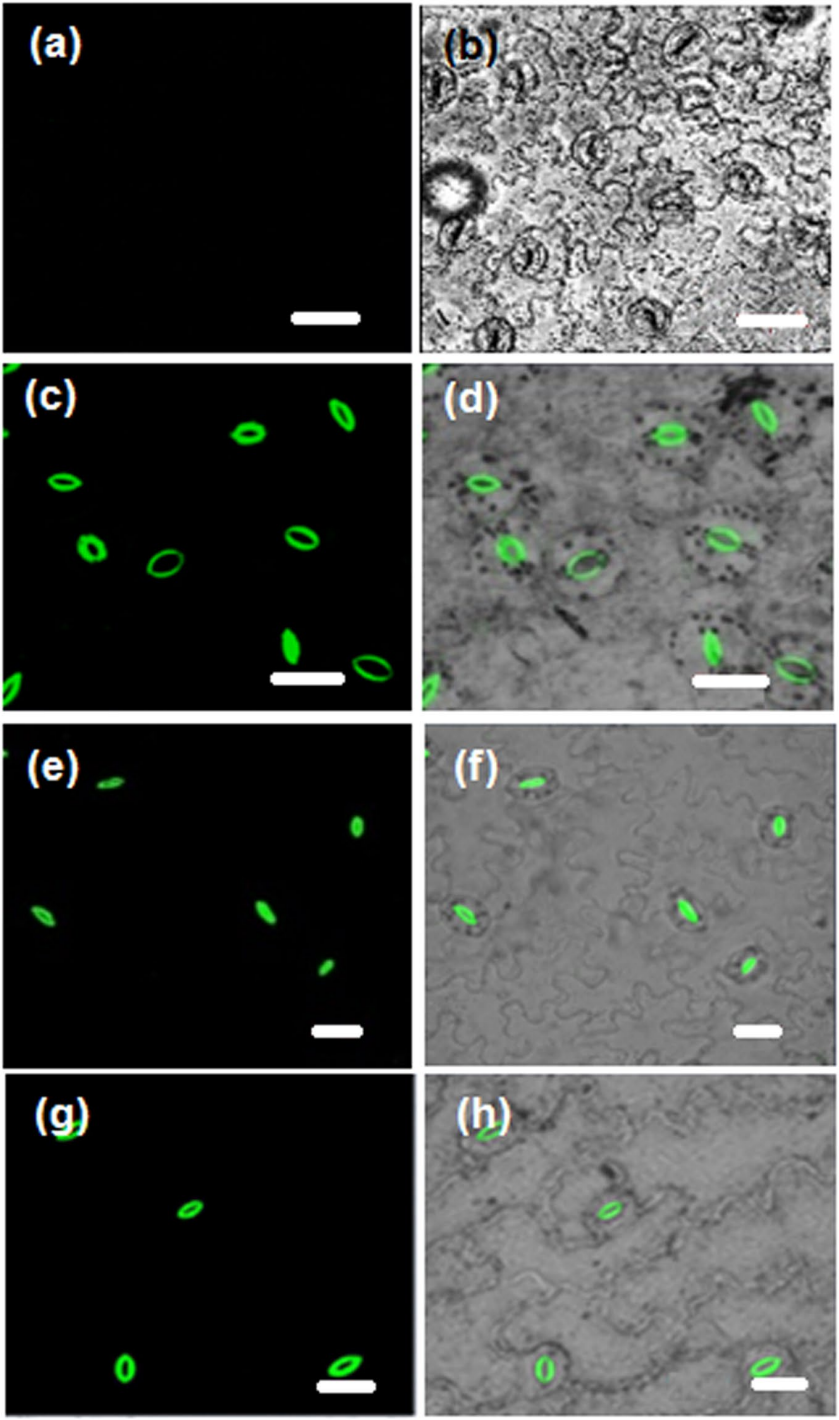
CLSM images. Images (fluorescence and overlay of fluorescence with bright field) of pea epidermis kept in buffer medium (500 µL): (**a**,**b**) control, and stained using BT_2_ solution in water (15 µL of 1 mM) under different conditions: (**c**,**d**) irradiated for 3 h and stained, (**e**,**f**) kept in the dark for 3 h and stained, (**g**,**h**) stained and irradiated for 3 h (excitation wavelength: 488 nm). Scale bar: 20 µm.

Imaging experiments were also conducted on the epidermal layer kept in 500 µL of a buffer solution in which BT_2_ solution in water (15 µL of 1 mM) has been added earlier (BT_2_ in DMSO gives similar results, Suppl. Fig. S1); it may be noted that the effective concentration of BT_2_ in this case is only 29 µM. After allowing the dye adsorption (for just 10 min), the sample was taken out, washed and placed on a microscope slide, protected with a cover slip and imaged in a CLSM. With the very low dye concentration used, only the inner guard cell wall is stained. Images obtained using BT_2_ in water, of the samples maintained under different conditions are shown in Fig. 4; the guard cells are closed in the samples kept in the dark and stained (Fig. 4e,f), but open in samples photo-irradiated and stained (Fig. 4c,d), as well as those that were photo-irradiated in presence of the dye (Fig. 4g,h). The latter images clearly prove that the staining process did not affect the stomatal opening; the related question of cytotoxicity is addressed at the end. Figure 3c,d shows the 3-D view of the open inner guard cell wall. It is clear that the staining process does not affect the stomatal opening. As the buffer helps to keep the cells alive, staining with BT_2_ is of potential interest in live-cell studies that target the dynamics of the cell wall or related phenomena. A comparison with dyes such as aniline blue, DAF-2DA, BCECF-AM, propidium iodide etc. commonly used for stomatal imaging shows that BT_2_ is advantageous in terms of the concentrations and time required for staining, as well as the solvent selections (Suppl. Table S1); in view of toxicity and environmental impact considerations, the possibility of using aqueous solutions is of major practical advantage, as noted earlier. Further, BT_2_ has the unique capability of selective staining of the walls and nuclei of the guard cells and epidermal cells by suitable variation of the medium and solvent.

Imaging experiments carried out with leaves of dicotyledon plants such as crape jasmine (*Tebernaemontana divaricate*), paper rose (*Bougainvillea glabra*), and thale cress (*Arabidopsis thaliana*) as well as a monocotyledon plant onion (*Allium cepa*) showed that BT_2_ is useful for a range of specimens (Suppl. Fig. S2). Experiments were conducted also with DPZDQ (Fig. 1) to explore the general utility of DADQs in imaging. Even though DPZDQ has limited solubility in water, good quality images could be obtained using aqueous as well as DMSO solutions (Suppl. Fig. S3). With the DMSO solution, it was noticed that the washed samples displayed reduced staining compared to the unwashed ones, suggesting partial removal of the dye. We have found that other DADQ derivatives with amine groups as in DPZDQ are also efficient for stomatal imaging; this should allow expanding the portfolio of DADQs for bioimaging applications.

**Figure 5:**
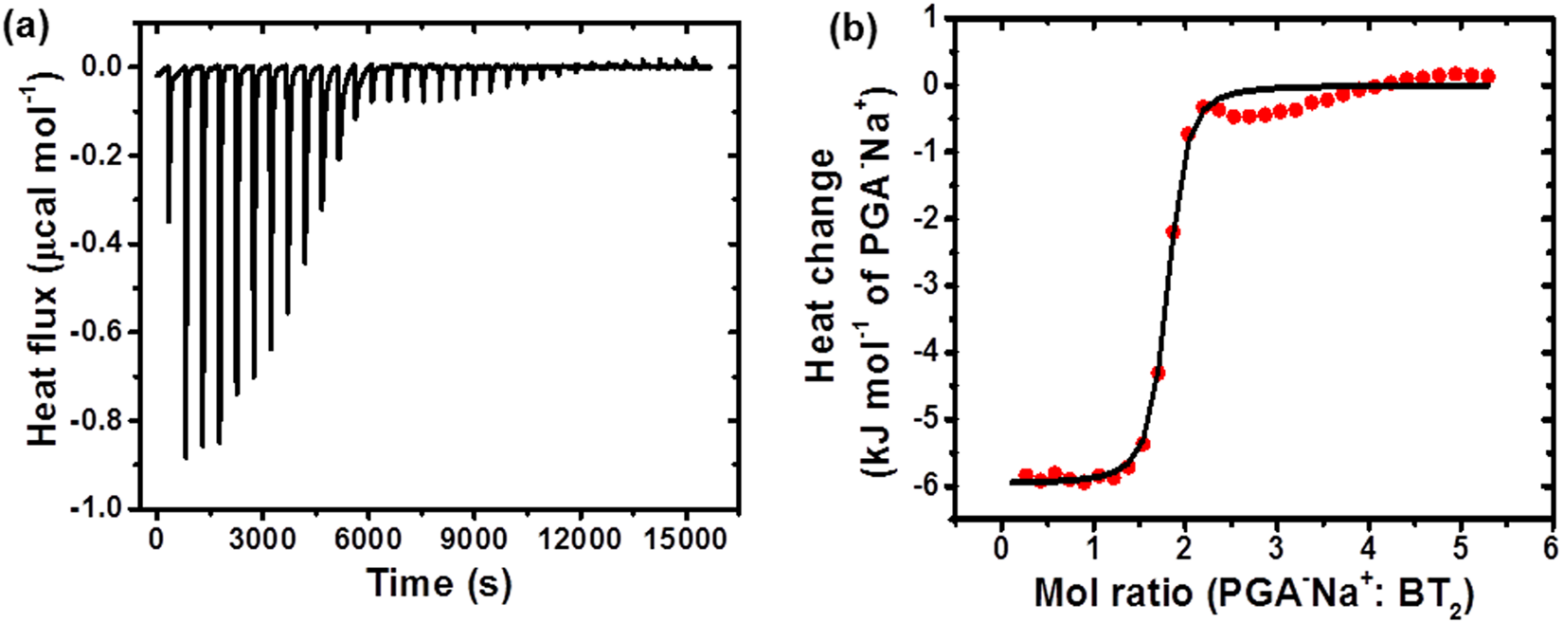
ITC data. (**a**) Raw and (**b**) integrated thermograms from the isothermal titration of PGA^−^Na^+^ into BT_2_ in aqueous solution. Fitting of the integrated thermogram is shown in (**b**).

**Figure 6:**
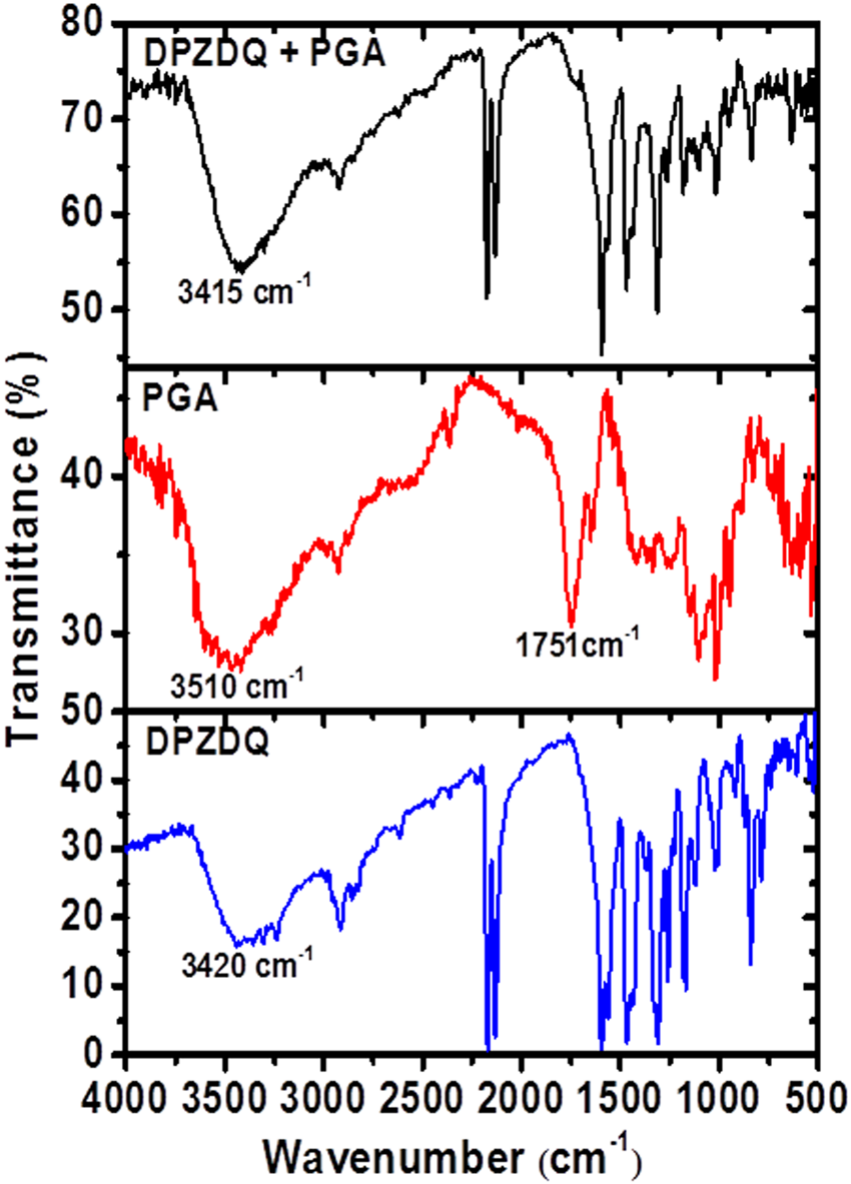
FTIR spectra. Spectra of DPZDQ, PGA and DPZDQ + PGA mixture; labeling of relevant peaks is indicated.

In order to gain insight into the molecular level interactions that facilitate the staining process, we have carried out control experiments with a few more selected DADQ derivatives. As seen above, BT_2_ (with the ionic piperazinium groups) and DPZDQ (with the H-bonding piperazine groups) stain the stomata; the stronger binding in the case of the former, suggested by the experiments on the washed samples, can be attributed to the electrostatic nature of the interactions. Experiments with DADQ derivatives bereft of both functionalities showed no effective staining (Suppl. Fig. S3). The dominant biomacromolecule in the cell wall, namely polygalacturonic acid (PGA), in neutral (carboxylic acid) as well as anionic (calcium salt of carboxylate) states, is likely to be a major target for molecular level interactions for BT_2_ and DPZDQ. In an effort to probe this point, isothermal titration calorimetry (ITC) experiments were carried out with BT_2_ in aqueous medium^36^, titrated against the sodium salt of PGA (PGA^−^Na^+^), also dissolved in water (concentration based on the lower bound of the assay was used in the analysis). The thermograms recorded are shown in Fig. 5; data analysis shows that the heat changes follow a simple binding model with an equilibrium constant of 1.33 × 10^6^ dm^3^ mol^−1^ and enthalpy and entropy changes of −5.97 kJ mol^−1^ and 97.1 J mol^−1^ K^−1^ (Suppl. Table S2). These are indicative of strong binding, effected through enthalpic and entropic contributions. The molar ratio for binding is found to be 1.75, close to that expected for the two piperazinium sites of BT_2_ locking with the negatively charged carboxylate sites on the PGA polyanion. ITC experiments with DPZDQ and PGA^−^Na^+^ (Suppl. Table S2, Fig. S4) indicated no meaningful binding; this may be attributed to the absence of any significant electrostatic interactions between the two. Due to problems of solubility in water, PGA in the fully neutral form could not be studied; organic media were not attempted, as it would be inappropriate for model studies relevant to processes in the biological system. In order to probe the possible molecular level interactions that could lead to the DPZDQ staining, we have recorded the Fourier transform infra-red (FTIR) spectra of DPZDQ, PGA and the mixture of the two prepared by grinding the solids together (Fig. 6). The prominent peak due to the carbonyl stretch vibration in PGA at ~1751 cm^−1^ is found to diminish significantly in the mixture; this is likely to be a consequence of the H-bonding interaction with the piperazine moieties in DPZDQ. It is noticed also that the broad peaks due to N-H stretch vibration in DPZDQ centered around 3420 cm^−1^ and O-H stretch vibration in PGA around 3510 cm^−1^, transform to a relatively narrower peak at 3415 cm^−1^ in the mixture, which again is suggestive of a well-defined H-bonding situation.

PGA in the leaf cell walls has both ionic (carboxylate) and neutral (carboxylic acid) sites. The well-known ‘egg box model’ envisions Ca^2+^ ions stitching the PGA chains together through the carboxylate groups^37^. The strong binding between PGA^−^Na^+^ and BT_2_ demonstrated by the ITC experiment, is enabled by the interaction of the dicationic moiety, B^2+^ with the ionic sites of PGA^−^. This would involve displacement of the Ca^2+^ ions as reported with propidium iodide using fluorescence imaging^38^; this possibility is further supported by the distance of 7.7 Å between the ammonium N atoms in the piperazinium moieties of BT_2_^34^, which fits well with the inter-chain distance of 6–8 Å in calcium pectate^39^. We visualized that a precise analysis of the local elemental composition, specifically in terms of the Ca content in the guard cell wall and regions such as the epidermal cell junctions could provide direct and critical insight into this; monitoring the composition of other ions like K^+^ which are not involved in the binding of the dye with the biomacromolecules would serve as useful control. The epidermal layer placed in pure water, as well as in aqueous solution of BT_2_ and subsequently washed, were imaged in a field emission scanning electron microscope (FESEM), and energy dispersive X-ray (EDX) spectra recorded at several points in the relevant regions; the images and representative spectra are shown in Fig. 7a,b. Average values of the atomic content of Ca and K in the inner guard cell wall region under different treatments of the epidermis including two concentrations of BT_2_, DPZDQ and propidium iodide are plotted in Fig. 7c (see also Suppl. Table S3). A clear reduction in the Ca content is seen in the samples treated with BT_2_, with the effect being enhanced with higher concentration of the dye; changes in the K content are not significant. DPZDQ has practically no impact as expected (Suppl. Fig. S5). Experiments with propidium iodide show effects similar to BT_2_. Parallel trends are seen in the epidermal cell junction as well (Suppl. Fig. S6); all these experiments used pure water medium for placing the samples, as the buffer medium itself was found to disturb the composition of Ca and K ions (Suppl. Table S4).

The above experiments establish the binding of DADQs with PGA in different forms. The staining pattern of the guard cell walls (inner and outer) and epidermal cell wall (Fig. 2) is a consequence of the dye concentration, and likely to be related to the PGA content in the different regions. With higher concentrations, the nuclei of the epidermal cells also get stained, as the epidermal cell walls are relatively thinner. The DMSO medium with a lower dielectric constant than water, allows easier passage of the BT_2_ salt into the guard cell leading to the staining of the nucleus. In the case of the nuclei, B^2+^ is likely to bind to the polyanions of the nucleic acids; it is notable that DPZDQ does not stain the nucleus.

**Figure 8:**
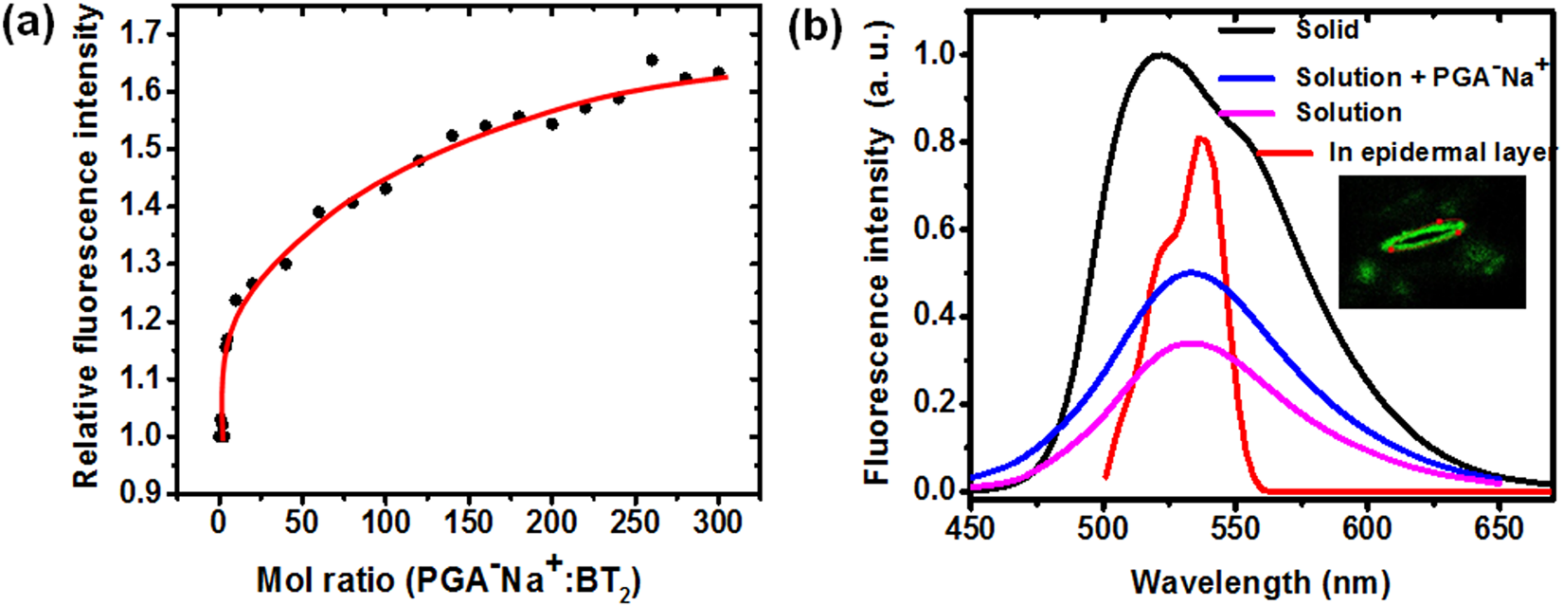
Fluorescence experiments. (**a**) Variation of the intensity of the fluorescence emission of BT_2_ on adding PGA^−^Na^+^ in increasing mol ratios (the line is only a guide to the eye); (**b**) fluorescence emission spectra of BT_2_ in different forms: as microcrystalline solid, aqueous solution, aqueous solution with PGA^−^Na^+^ (200:1 ratio) and stain in pea epidermal layer (recorded in CLSM at the red points shown in the image in the inset).

Having gained an understanding of the molecular level interactions responsible for the dye binding with the cell wall or organelles, we have probed the basis for the strong fluorescence that facilitates the bright and high contrast imaging. As noted earlier, the DADQs show enhanced emission in the aggregated state due to the restriction of internal motions as well as the obstruction of intermolecular energy transfer pathways^11, 16^; the former would indeed be relevant in highly viscous and rigid environments^36^. In order to mimic the impact of the binding of BT_2_ and DPZDQ in the cell wall, we have monitored its fluorescence response in aqueous medium, in presence of increasing amount of PGA^−^Na^+^; relevant spectral data are presented in Suppl. Figs S7, S8. As the polymer: BT_2_ mol ratio (see Methods section for details) increases, the fluorescence intensity increases and begins to saturate above a ratio of ~200:1 (Fig. 8a); the λ_max_ remains nearly constant at ~535 nm as in the pure aqueous solution (Fig. 8b). These observations suggest that the interaction between the dye and the polymer and the local viscosity due to the polymer chains reduce the internal motions of the dye molecule, leading to fluorescence enhancement. The emission spectrum recorded on the CLSM corresponding to the fluorescence image of the stomatal guard cell stained with BT_2_ (Fig. 8b) shows a λ_max_ ~ 537 nm with a shoulder at ~525 nm. The blue shifted peak is indicative of the presence of neighboring zwitterionic BT_2_ molecules, and their local field effects^15^. This is supported by the emission spectrum of BT_2_ in the microcrystalline solid with a λ_max_ at ~522 nm (Fig. 8b); the broadening arises due to different intermolecular interactions. It should be noted that the emission intensity is enhanced significantly more in the solid^11^; the spectrum in the figure is meant only to highlight the peak positions. The various observations suggest that the BT_2_ molecules exist in isolated (as in solution, but in a rigid environment) as well as aggregated (as in the solid) states within the cell wall; similar situation is likely in the case of nucleus staining as well. The strong fluorescence emission of BT_2_ bound within the confines of the cell can be attributed to the restriction of excited state geometry relaxations as well as local aggregation effects.

As various biological systems exhibit autofluorescence, it is pertinent to verify the relevance of BT_2_ based imaging in the current context. Autofluorescence of the guard cell wall arises due to components such as ferulic acid, *p*-coumaric acid and cinnamic acid present; pattern, color and intensity vary with the species. Cell walls of the pea epidermis layer are known to show relatively weak autofluorescence compared to other species^40, 41^. We have recorded the autofluorescence response of the pea leaf epidermal cells in the CLSM by exciting at different wavelengths: 514, 488 and 405 nm. The images obtained even with significantly higher laser power and gain, are quite dull compared to those obtained with BT_2_ based imaging (Suppl. Figs S9A, S10A) highlighting the utility of the latter; the enhancement of brightness is quantified by the fluorescent intensity profiles (Suppl. Figs S9B, S10B). We have also probed the general utility of DADQs as bio-imaging dyes, by carrying out preliminary experiments on mammalian and bacterial cells; the observations confirm that they are potentially useful for imaging different kinds of cells (Suppl. Figs S11, S12). Imaging specific organelles or proteins is a critical issue; as DADQs are amenable to derivatization with a wide range of functional groups, this should indeed be possible. For example, the 6-((4-aminomethyl)benzyl)oxy)-9*H*-purin-2-aminium) derivative may be synthesized using the standard protocol and used as a substrate for the SNAP tag; similar constructs can be generated for tags such as CLIP, HALO, ACP and MCP. The protein-protein interactions are likely to enhance specificity and the covalent functionalization the strength of the binding, over the less discriminating and relatively weaker H-bond or electrostatic interactions. Finally, we have examined several factors relevant to the practical use of BT_2_ and DPZDQ. They have relatively high melting points and are stable for extended periods of time under ambient conditions requiring no specialized conditions for storage; they also show very good photo-stability (Suppl. Fig. S13). Another important issue related to the application of the new dye in bioimaging is its cytotoxicity, that could be detrimental to the cells being imaged and also make the handling hazardous. (3-(4,5-dimethylthiazol-2-yl)-2,5-diphenyltetrazolium bromide) tetrazolium (MTT) assay experiments using L929 cell lines (normal cell) indicated that both BT_2_ and DPZDQ are absolutely non-toxic (cell viability data are provided in Suppl. Fig. S14). Experiments using HeLa cell lines also showed that they have no cytotoxicity; the IC_50_ values of BT_2_ and DPZDQ are found to be 1468 ± 345.4 µg/ml and 790.70 ± 98.73 µg/ml respectively (Suppl. Fig. S15). All these factors highlight the practical utility as well as efficiency of BT_2_ and other DADQs in bioimaging.

## Conclusions

We have demonstrated the efficient use of DADQs with appropriate functionalities in fluorescence based stomatal imaging. Selective staining of cell walls or cell walls with the nuclei could be achieved; the latter is particularly relevant in view of the fact that many of the traditionally used dyes are not selective, or stain only the cell wall. In addition to the bright and high contrast images that they provide, the simple and flexible molecular design and low cost of DADQs are important features. Calorimetry, microscopy and spectroscopy based investigations provide useful insight into the molecular level interactions involved in the binding of the dye molecules to the biological system and the basis for the enhanced fluorescence responses. DADQs are potentially efficient fluorescence labels that can be incorporated through non- covalent interactions for a variety of wider bioimaging applications.

## Methods

### Materials

DPZDQ and BT_2_ were synthesized using reported procedure, by the reaction of tetracyanoquinodimethane with piperazine followed by salt formation with *p*-toluene sulfonic acid^34, 35^; they were purified by recrystallization and characterized (see Supplementary Information). Polygalacturonic (pectic) acid (PGA, assay ≥90%) and its sodium salt (PGA^n−^ nNa^+^, abbreviated as PGA^−^Na^+^, Assay ≥75%) purchased from Aldrich Chemicals were used as such. 2-(*N*-morpholino)ethanesulfonic acid (MES) buffer was prepared as follows: MES (Aldrich Chemicals) was dissolved in high purity water (Millipore MilliQ, Resistivity = 18 MΩ cm), the pH was adjusted to 7.0 by adding potassium hydroxide, and potassium chloride was added to form the final solution (10 mM in MES-KOH and 50 mM in KCl). All experiments were carried out using high purity water.

### Specimen preparation and imaging

Epidermal strips were carefully pealed from the abaxial surface of the leaves of 2–3 weeks old pea plant grown in a greenhouse at 25 °C with supplementary lighting, washed with water and cut into ~4 × 4 mm^2^ pieces. The strips were placed on a microscope slide, stained directly with the appropriate dye solution, washed by brief dipping in water, protected with a cover slip, and imaged. Alternately, the strips were placed in the MES buffer in a 6-well plate and maintained either in the dark or illuminated with a 100 W tungsten lamp with water jacket protection. The specimen was then transferred to a 24-well plate containing the MES buffer with the required dye solution, washed, placed on a microscope slide, protected with a cover plate and imaged. A Leica model ZEISS LSM 880 confocal laser scanning microscope (CLSM) was used for imaging, and an Ar/Ar-Kr laser as the excitation (488 nm) source; emission in the 500–650 nm range was detected. Images were obtained using 60x and 40x objective lens. Parameters like the detector gain and amplification offset/gain were adjusted to optimize the fluorescence intensity of the targets and the background. Under these experimental conditions the dyes are found to be quite photo-stable. Results of control experiments without the dyes, under identical settings are added in Suppl. Fig. S3. Images were processed using ZEN software.

### Isothermal calorimetry

A Microcal Model VP-ITC isothermal titration calorimeter (ITC) was used. All studies were carried out at 298 K. A 4 mM aqueous solution of PGA^−^Na^+^ was titrated into a 125 µM aqueous solution of BT_2_ taken in the cell; water was used in the reference cell. A control experiment, in which PGA^−^Na^+^ was injected into pure water in the cell, was used to correct the measured heats for dilution effects. Origin 7.0-based software was used for data analysis.

### Electron microscopy

Field emission scanning electron microscope (FESEM) images of the epidermal layer (with and without staining, and dried) placed on a silicon wafer and provided with a thin coating of sputtered gold, was recorded on a Carl Zeiss model Merlin Compact 6027 FESEM with a beam voltage of 10.0 kV. Energy dispersive X-ray (EDX) spectra were recorded using an Oxford Instruments X-Max^N^ SDD (50 mm^2^) system and INCA analysis software; atomic compositions were estimated by averaging data from typically six samples with spectra collected at 4–5 different points in each case.

### Infra-red spectroscopy

Fourier transform infra-red (FTIR) spectra of samples prepared in the form of KBr pellets, were recorded using a Thermo Scientific model Nicolet 380 FTIR spectrometer.

### Fluorescence experiments

0.5 mL of 0.2 mM aqueous solution of BT_2_ was taken in a quartz cuvette and appropriate volumes of a 10 mM solution of PGA^−^Na^+^ were added to obtain varying mol ratios (polymer: BT_2_; calculated using the monomer molecular weight, taking into account the purity of the polymer). The solution was mixed thoroughly and made up to 3 ml in each case, ensuring identical concentration of BT_2_ in all experiments. Absorption and fluorescence emission spectra were recorded on a Varian model Cary 100 UV-Vis spectrometer and Horiba Jobin Yvon model FL3-22 Fluorolog spectrofluorimeter respectively. Absorbance of BT_2_ was the same in all the samples. Fluorescence intensities were calculated by the integration of the spectra plotted as a function of wavenumber. The fluorescence quantum yield of solutions were determined by comparison with quinine sulfate solution in 1 N H_2_SO_4_ (φ = 0.546); absolute quantum yield of the solids was measured using an integrating sphere. Average excited state lifetime of the solids was determined using a time-resolved confocal fluorescence microscope (MicroTime 200, PicoQuant) coupled to an Olympus IX71 microscope (PicoQuant). Excitation was carried out using 485 nm pulsed-laser diodes and the fluorescence observed through 510 nm long-pass filters respectively; the corresponding fwhm of pulse response functions was 144 ps. Data acquisition was performed with a PicoHarp 300 TCSPC module using PicoHarp300 version 2.3 in a time-tagged time-resolved mode.

### Data Availability

The datasets generated during and/or analysed during the current study are available from the corresponding author on reasonable request.

## Electronic supplementary material

Supplementary Information
