# Supplementary material for: Efficient Bioimaging with Diaminodicyanoquinodimethanes: Selective Imaging of Epidermal and Stomatal Cells and Insight into the Molecular Level Interactions
Source: Sci Rep. 2017 Sep 6;7:10583. doi: 10.1038/s41598-017-11293-y (PMC5587692; doi:10.1038/s41598-017-11293-y)
Supplement: Supplementary file 1 — Supplementary Information [file 41598_2017_11293_MOESM1_ESM.pdf]

Supplementary Information for

Efficient Bioimaging with Diaminodicyanoquinodimethanes: Selective Imaging of Epidermal and Stomatal Cells and Insight into the Molecular Level Interactions

N. Senthilnathan, Ch. G. Chandaluri and T. P. Radhakrishnan*

Details of characterization of the dyes, confocal laser scanning microscopy studies of pea stomata, different leaf stomata, mammalian and bacterial cells, autofluorescence analysis of pea epidermal cells, isothermal titration calorimetry, infra-red spectra, FESEM, EDX analysis, absorption and fluorescence spectra, photo-stability studies and cytotoxicity assay.

Page **Contents**

Page	Contents
S2	Characterization of BT_2 and DPZDQ
S3-S4	Comparison with other dyes (Table S1)
S5	Imaging of pea stomata with BT_2 in DMSO (Fig. S1)
S6	Imaging of different leaf stomata (Fig. S2)
S7	Comparison of CLSM images (Fig.S3)
S8	Isothermal titration calorimetry (Table S2, Fig. S4)
S9-S10	FESEM, EDX analysis (Fig. S5, S6, Tables S3, S4)
S11	Absorption and fluorescence spectra (Fig. S7, S8)
S12	Autofluorescence analysis of pea epidermal cells (Fig. S9, S10)
S13	Imaging of mammalian and bacterial cells (Fig. S11, S12)
S14	Photo-stability studies (Fig. S13)
S15-S18	Cytotoxicity assay (Fig. S14, S15)

Characterization of BT_2 and DPZDQ

BT_2

Yield= 75%, recrystallized from acetonitrile; m.p. 310ºC (dec.); FTIR (KBr): $\bar{\nu}$/cm^{-1}= 2177.4, 2138.6, 1136.1; ^{1}H NMR (d_6-DMSO): δ/ppm= 9.0 (s, 4H), 7.49 (d, 4H), 7.38 (d, 2H), 7.12 (d, 4H), 6.88 (d, 2H), 3.8 (m, 4H), 3.46 (m, 4H), 2.2 (s, 6H); ^{13}C NMR (d_6-DMSO): δ/ppm = 21, 36, 41, 43, 48, 113, 118, 123, 125, 128, 132, 138, 139, 145, 150, 171; elemental analysis (calcd., found for $BT_2.H_2O$ i.e. $C_{32}H_{40}N_6O_7S_2$) : %C = (56.23, 56.14), %H = (5.82, 5.85), %N = (12.15, 12.28). λ_{max}^{abs}: 415 nm, λ_{max}^{emi}: 538 nm, Stokes shift: 123 nm, quantum yield: 14.2 % (solid state), 0.05 % (solution state), lifetime: 0.63 ns, ε: 10,912 $M^{-1}cm^{-1}$, brightness: 154.942 $M^{-1}cm^{-1}$.

DPZDQ

Yield= 80%, recrystallized from DMF-ether; m.p. 300ºC (dec.); FTIR (KBr): $\bar{\nu}$ /cm^{-1}=2171.6, 2130.3, 1593.1; ^{1}H NMR (d_6-DMSO): δ/ppm=7.3 (d, 2H), 6.8 (d, 2H), 3.5 (s, 2H), 3.3-3.1 (m, 8H), 2.8 (m, 8H); ^{13}C NMR (d_6-DMSO): δ/ppm = 168, 149, 132, 123, 118, 114, 53, 46, 34; elemental analysis (calcd., found for $C_{18}H_{22}N_6$) : %C = (67.15, 67.8), %H = (6.81, 6.83), %N = (26.15, 26.08). λ_{max}^{abs}: 391 nm, λ_{max}^{emi}: 522 nm, Stokes shift: 131 nm, quantum yield: 5.8 % (solid state), 0.07 % (solution state), lifetime: 0.75 ns, ε: 17,871 $M^{-1}cm^{-1}$, brightness: 103.652 $M^{-1}cm^{-1}$.

Comparison with other dyes

Table S1. Comparison of commercial dyes used for stomatal imaging with BT_2 in terms of conditions and selectivity of staining.

Dye	Solvent	Concentration (mM)	Staining time (min)	Part stained	Ref.
**Sample in buffer**					
Aniline Blue	Water	0.67	-	Cell wall	S1
Aniline Blue	Water	0.67	-	Cell wall	S2
DAF-2DA#	DMSO	0.02	30	Full cell	S3, S4
DAF-2DA#,*	DMSO	0.01	30	Full cell	S5
BCECF-AM#,*	DMSO	0.005	30	Full cell	S5, S3
H2DCF-DA#,*	Ethanol	0.03	30	Full cell	S5
BT_2	Water, DMSO	0.029	10	Inner cell walls	**This work**
**Sample in buffer-free state**					
PI#	Water	1.5	2	Epidermis cell wall	S6
PI#	Water	0.03	1-5	Cell wall	S7
PI#	Water	0.02	5	Cell wall of viable cells	S8
Safranin	Ethanol-water	28.5	-	Cell wall	S9
Safranin	Water	28.5	-	Cell wall	S10
Safranin	Ethanol-water	28.5	-	Cell wall	S11
Safranin	Water	28.5	4 - 8	Cell wall	S12
BT_2	Water, DMSO	1.0	2	Cell walls / nucleus	**This work**

*Dark state required.

#DAF-2DA : 4,5-diaminofluorescein diacetate; BCECF-AM: 2′,7′-bis(2-carboxyethyl)-5(6)-carboxyfluorescein acetoxymethyl ester; H2DCF-DA: 2′,7′-dichlorofluorescein diacetate; PI: propidium iodide

References

S1. Apostolakos, P., Livanos, P. & Galatis, B. Microtubule involvement in the deposition of radial fibrillar callose arrays in stomata of the fern Asplenium nidus L. *Cell Motil. Cytoskel.* **66,** 342–349 (2009).

S2. Apostolakos, P., Livanos, P., Nikolakopoulou, T. L. & Galatis, B. Callose implication in stomatal opening and closure in the fern Asplenium nidus. *New Phytol.* **186,** 623–635 (2010).

S3. Puli, M. R. *et.al.* Stomatal closure induced by phytosphingosine-1-phosphate and sphingosine-1-phosphate depends on nitric oxide and pH of guard cells in *Pisum sativum. Planta* **244,** 831–841 (2016).

S4. Agurla, S., Gayatri, G. & Raghavendra, A. S. *Plant Nitric Acid Methods and Protocols* (ed. Gupta, K. J.) 49-56 (Humana Press, 2016).

S5. Puli, M. R. & Raghavendra, A. S. Pyrabactin, an ABA agonist, induced stomatal closure and changes in signalling components of guard cells in abaxial epidermis of Pisum sativum. *J. Exp. Bot.* **63,** 1349–1356 (2012).

S6. Kong, Z., Hotta, T., Lee, Y.-R. J., Horio, T. & Liu, B. The γ-tubulin complex protein gcp4 is required for organizing functional microtubule arrays in arabidopsis thaliana. *Plant Cell* **22,** 191–204 (2010).

S7. Hachez, C., Ohashi-Ito, K., Dong, J. & Bergmann, D. C. Differentiation of arabidopsis guard cells: analysis of the networks incorporating the basic helix-loop-helix transcription factor, FAMA. *Plant Physiol*. **155,** 1458–1472 (2011).

S8. Chitrakar, R. & Melotto, M. Assessing stomatal response to live bacterial cells using whole leaf imaging. *J. Vis. Exp.* **44,** e2185 1–4 (2010).

S9. Tripathi, S. & Mondal, A. K. Taxonomic diversity in epidermal cells (stomata) of some selected anthophyta under the order leguminales (caesalpniaceae, mimosaceae & fabaceae) based on numerical analysis: a systematic approach. *Int. J. Sci. Nat*. **3,** 788–798 (2012).

S10. Ajuru, M. G. & Okoli, B. E. Morphological and Epidermal Studies on Certain Species of Napoleona P. Beauv. (Lecythidaceae) in Nigeria. *Int. J. Mod. Botany* **2,** 115–119 (2012).

S11. Tripathi, S. & Mondal, A. K. Comparative (quantitative and qualitative) studies of stomata of selected six medicinally viable species of cassial. *Int. J. Life Sci. Biotechnol. Pharma Res.* **1,** 104–113 (2012).

S12. Mitra, S., Maiti, G. G. & Maity, D. Structure and distribution of heteromorphic stomata in Pterygota alata (Roxb.) R. Br. (Malvaceae, formerly Sterculiaceae). *Adansonia* **37,** 139–147 (2015).

Imaging of pea stomata with BT_2 in DMSO

Images of samples in the buffer medium stained by BT_2 in DMSO, without and with irradiation; staining of the inner guard cell wall and the response to light are clearly observed again.

Figure S1. CLSM fluorescence images (at different magnifications) of pea epidermis kept in buffer medium (500 µL) and stained with BT_2 solution in DMSO (15 µL of 1 mM) under different conditions: **(a, b)** irradiated for 3 h and stained, **(c, d)** kept in the dark for 3 h and stained. Scale bars: (a), (c) = 20 µm; (b), (d) = 10 µm.

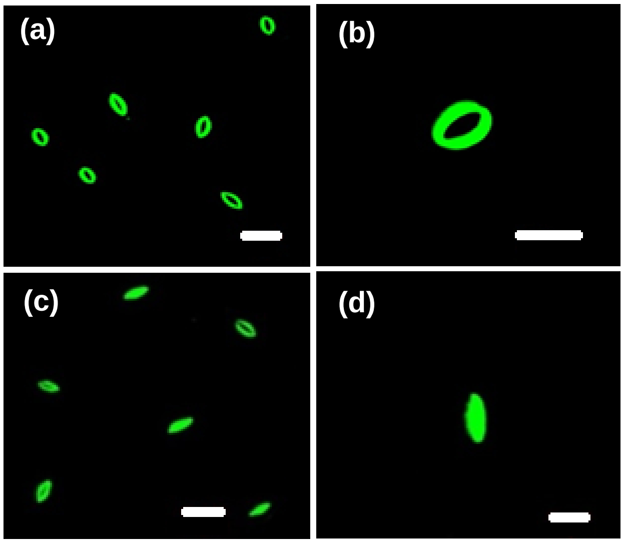

Imaging of different leaf stomata

Figure S2. CLSM images of stomata in the leaves of dicotyledon plants, **(a)** crape jasmine (*Tabernaemontana divaricate*), **(b)** paper rose (*Bougainvillea glabra*), and **(c)** thale cress (*Arabidopsis thaliana*), as well as a monocotyledon plant, **(d)** onion (*Allium cepa*) maintained in 500 µL buffer and stained using 15 µL aqueous solution of BT_2 having concentrations, 5 mM (a-c) and 0.05 mM (d). Scale bar: 20 µm.

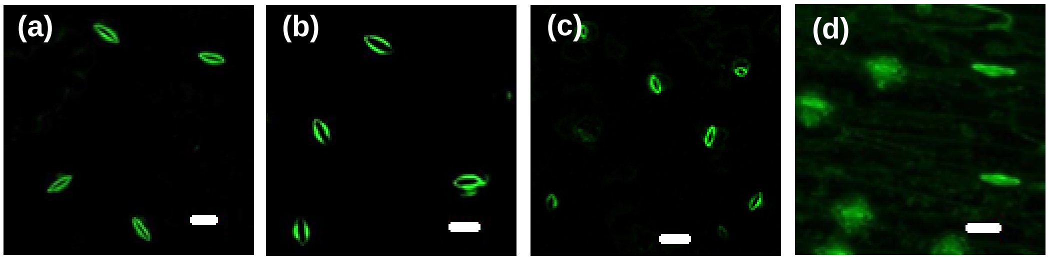

Imaging experiments carried out with leaves of dicotyledon plants showed that BT_2 is useful for a range of specimens.

Comparison of CLSM images

Figure S3. Comparison of CLSM images of pea epidermis in buffer-free state, stained using 15 µL of 1.75 mM solutions of different DADQ derivatives (structures shown) in water and DMSO for 2 min, with and without washing. Images recorded for control samples without the dye under identical microscope settings are also shown. Scale bar: 20 µm.

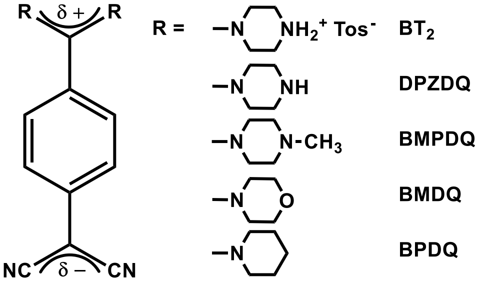

Dye	In water		In DMSO		Control
	No wash	With wash	No wash	With wash	
BT_2	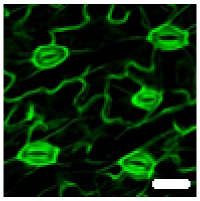	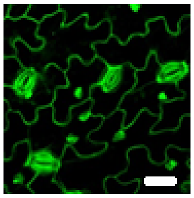	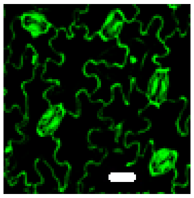	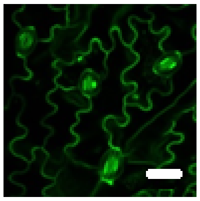	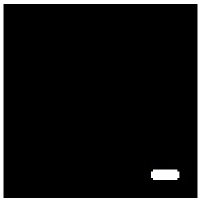
DPZDQ	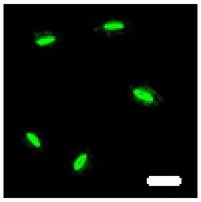	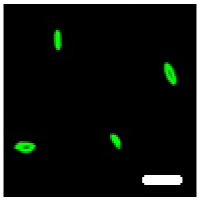	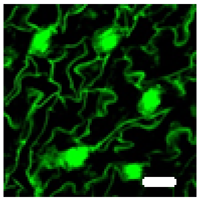	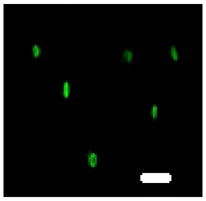	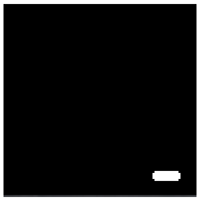
BMPDQ	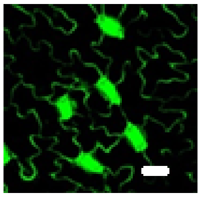	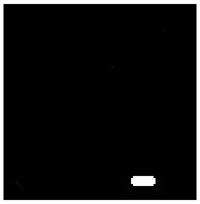	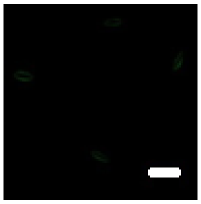	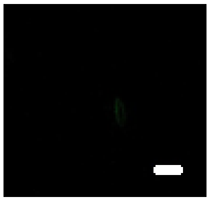	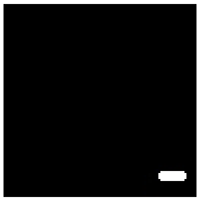
BMDQ	Insoluble	Insoluble	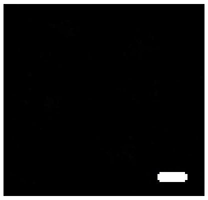	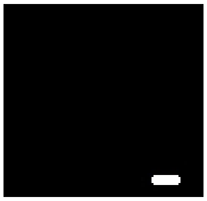	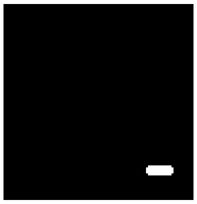
BPDQ	Insoluble	Insoluble	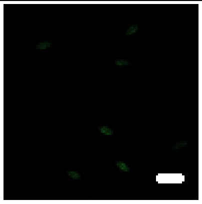	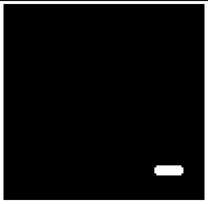	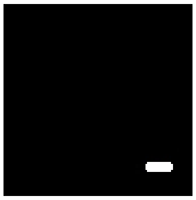

Isothermal titration calorimetry

Table S2. Binding and thermodynamic parameters from the ITC experiments on BT_2 and DPZDQ with $PGA^{-}Na^{+}$; errors are shown in parenthesis.

Parameter	Value (error)	
	**$PGA^{-}Na^{+}$: BT_2**	**DPZDQ : $PGA^{-}Na^{+}$**
N	1.74 (± 0.012)	0.117 (± 0.15)
K (10^5 $dm^3 mol^{-1}$)	13.3 (± 2.9)	0.116 (± 0.016)
ΔH^o (kJ mol^{-1})	-5.970 (± 0.0741)	-161.5 (± 217.2)
ΔG^o (kJ mol^{-1})	-34.9	-23.2
ΔS^o (J mol^{-1} K^{-1})	97.1	-464

Errors in the parameters determined for the DPZDQ / $PGA^{-}Na^{+}$ are very large; the data and the fitting are therefore not meaningful.

Figure S4. **(a)** Raw and **(b)** integrated thermograms from the isothermal titration of DPZDQ (2 mM) into $PGA^{-}Na^{+}$ (75 µM) in aqueous solution. Fitting of the integrated thermogram is shown in (b).

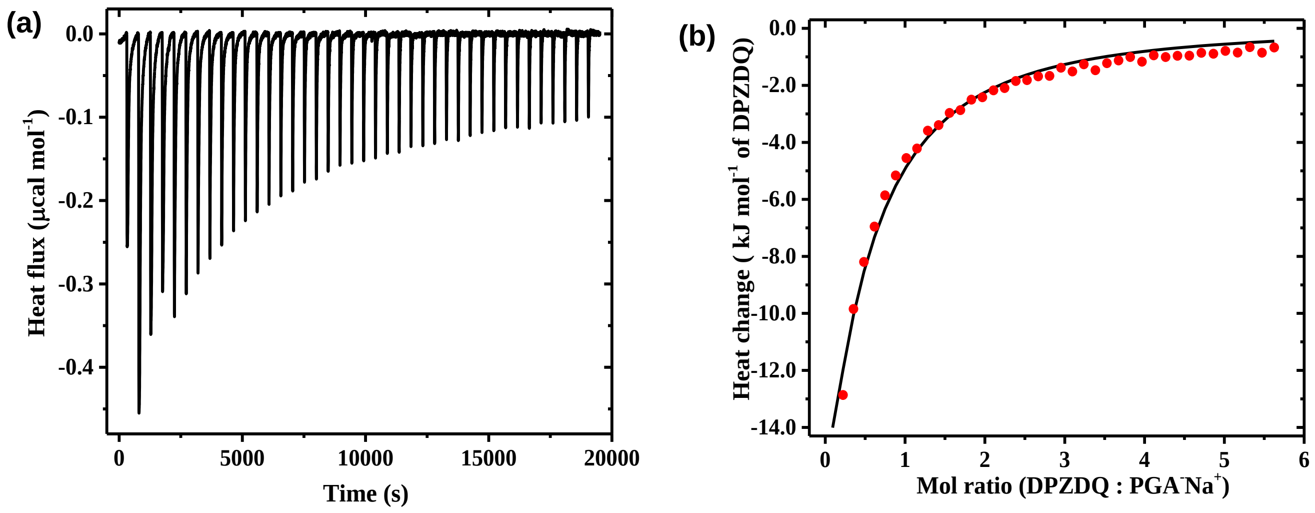

FESEM, EDX analysis

Figure S5. FESEM image and EDX spectra of selected regions (ECJ: epidermal cell junction; IGCW: inner guard cell wall) shown in the image, of pea epidermis stained using DPZDQ solution in water (0.5 mL of 5 mM) for 2 min.

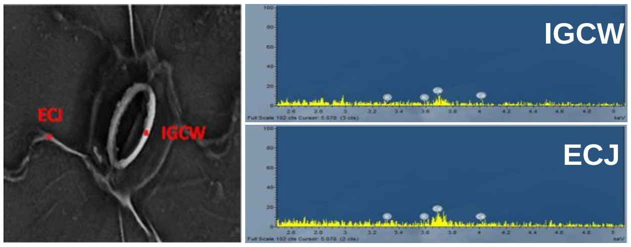

Figure S6. Average Ca and K content in the ECJ region of the pea epidermis under different conditions.

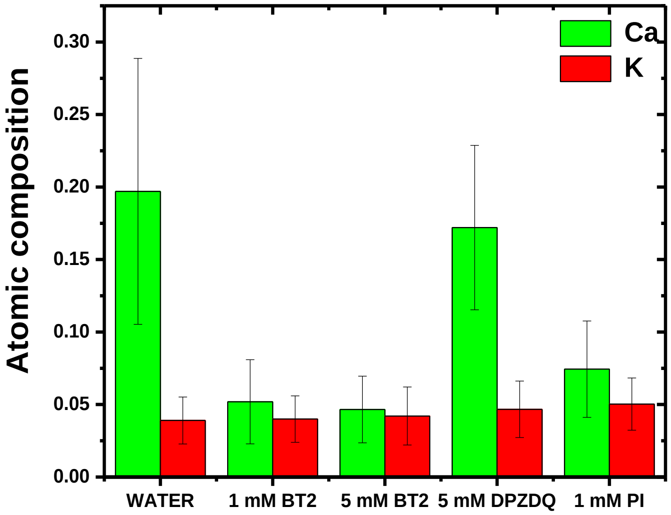

Table S3. Atom % of Ca and K (with standard deviations) on the inner guard cell wall (IGCW) and epidermal cell junction (ECJ) regions (Figure 7a, b of main text, Figure S5) of fresh (in water) and stained epidermis layer of pea plant leaf, determined using EDX spectroscopy with FESEM; dyes used are BT_2, DPZDQ, propidium iodide.

Sample treatment	IGCW		ECJ	
	**Ca**	**K**	**Ca**	**K**
Water	**0.122** (0.0568)	**0.0595** (0.0315)	**0.197** (0.0917)	**0.039** (0.0162)
BT_2 (1 mM in water)	**0.0549** (0.0358)	**0.0438** (0.0220)	**0.0519** (0.0290)	**0.0400** (0.016)
BT_2 (5 mM in water)	**0.0406** (0.0222)	**0.0544** (0.032)	**0.0466** (0.0230)	**0.0421** (0.020)
DPZDQ (5 mM in water)	**0.121** (0.0610)	**0.056** (0.035)	**0.172** (0.0567)	**0.0467** (0.0195)
Propidium iodide (1 mM in water)	**0.0500** (0.0330)	**0.0555** (0.0277)	**0.0744** (0.0332)	**0.0503** (0.0180)

Table S4. Atom % of Ca and K (with standard deviations) on the inner guard cell wall (IGCW) and epidermal cell junction (ECJ) regions, of epidermis layer of pea plant leaf maintained in water, MES buffer and MES buffer containing BT_2.

Sample treatment	IGCW		ECJ	
	**Ca**	**K**	**Ca**	**K**
Water	**0.122** (0.0568)	**0.0537** (0.0409)	**0.197** (0.0917)	**0.0445** (0.0345)
MES buffer (pH=7.0)	**0.0475** (0.0378)	**0.186** (0.111)	**0.173** (0.118)	**0.525** (0.173)
BT_2 (1 mM in water + MES buffer)	**0.0180** (0.00920)	**0.219** (0.0708)	**0.093** (0.0450)	**0.731** (0.162)

In the MES buffer, Ca^{2+} ions appear to leach out of the epidermis, and K^+ to move in.

Absorption and fluorescence spectroscopy

Absorption and fluorescence Spectra:

Figure S7. **(a)** Absorption and **(b)** fluorescence spectra of BT_2 and DPZDQ.

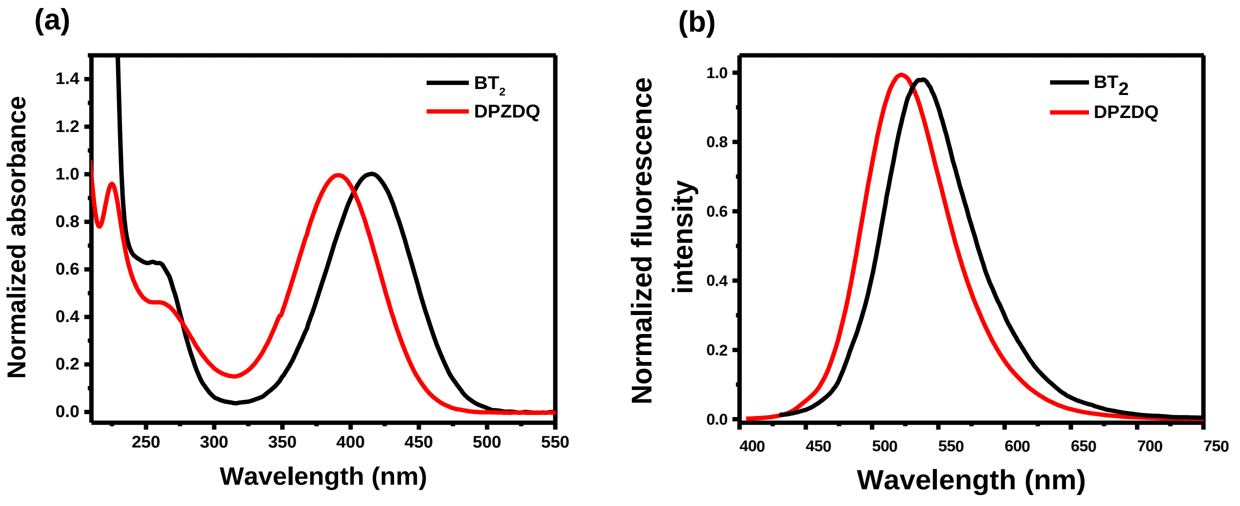

Relative changes of the spectral properties of BT_2 on adding PGA^-Na^+

As the polymer: BT_2 mol ratio increases, the fluorescence intensity increases and begins to saturate above a ratio of ~ 200:1.

Figure S8. (a) Absorption and **(b)** fluorescence spectra of BT_2-PGA^-Na^+ from mole ratio 1:1 to 1:300.

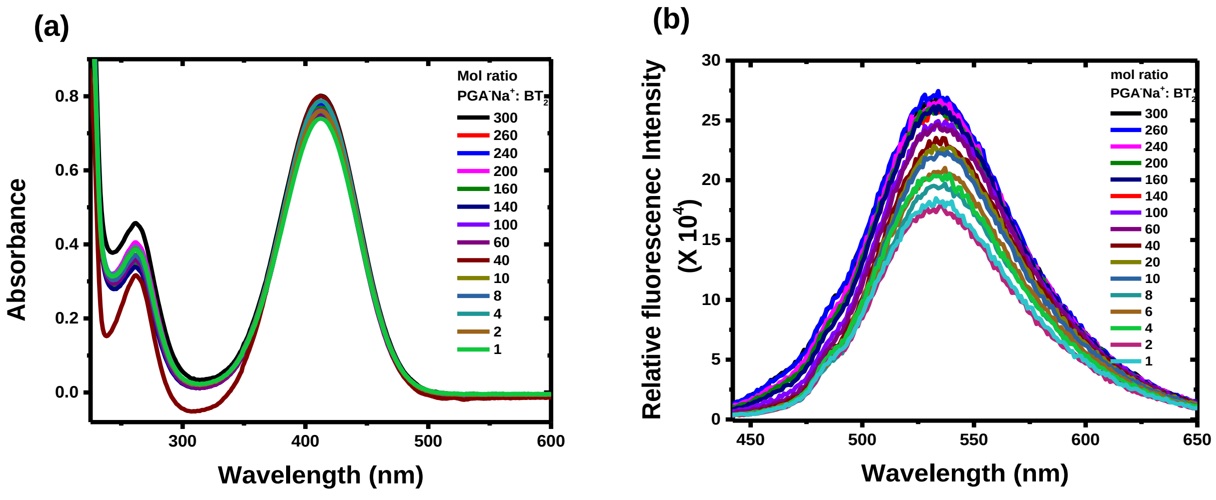

Autofluorescence analysis of pea epidermal cells

The autofluorescence of the pea epidermal cells was monitored under CLSM at different wavelengths, laser power and gain. The images recorded are shown in Figure S9.

Figure S9. (A) CLSM images using autofluorescence of fresh epidermis layer: (a) excitation wavelength : 514 nm, emission range: 540-600 nm, laser power: 100%, master gain : 1077 V, (b) excitation wavelength : 488 nm, emission range: 490-570 nm laser power: 100%, master gain : 940 V, (c) bright field, (d) excitation wavelength : 405 nm, emission range: 410-470, laser power: 35%, master gain : 881 V, (e) overlay of fluorescence with bright field. Scale bar: 20 µm. **(B)** Fluorescence intensity profiles [along the yellow lines marked on the stomata in the images in (**Aa, Ab, Ad**)].

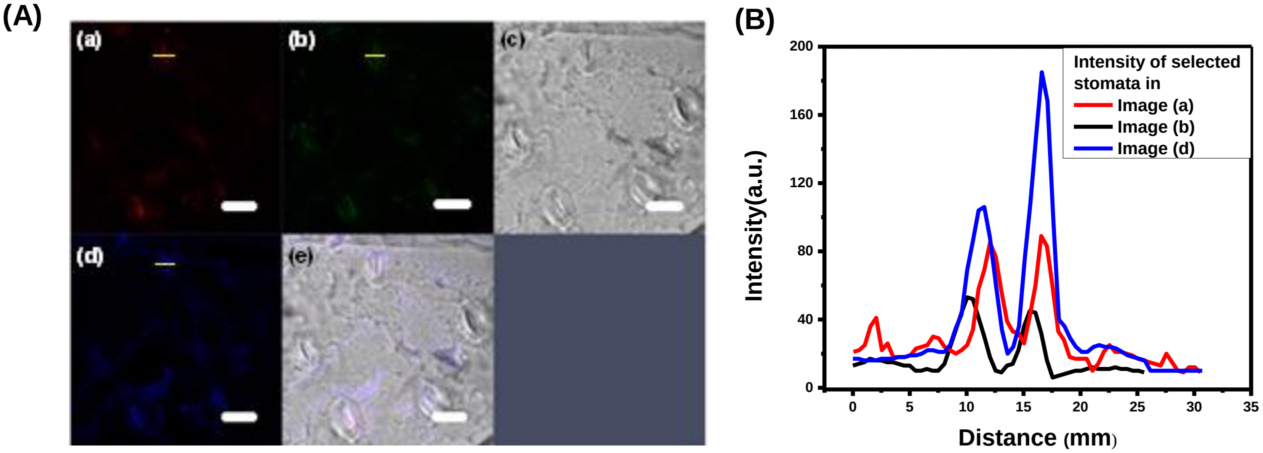

The contrasting images of stomata stained by BT_2 are shown in Fig. S10. It is seen that the autofluorescence images obtained even with significantly higher laser power and gain, are quite dull compared to those obtained with BT_2 based imaging.

Figure S10. (A) CLSM Images (fluorescence and overlay of fluorescence with bright field) of pea epidermis kept in buffer medium (500 µL): **(a, b)** control, and **(c, d)** stained using BT_2 solution in water (15 µL of 1 mM); excitation wavelength: 488 nm, emission range: 490-570 nm, laser power: 5% and master gain: 700 -800 V. Scale bar: 20 µm. **(B)** Fluorescence intensity profile [along the yellow line marked on the stomata in the image in (**Ac**)].

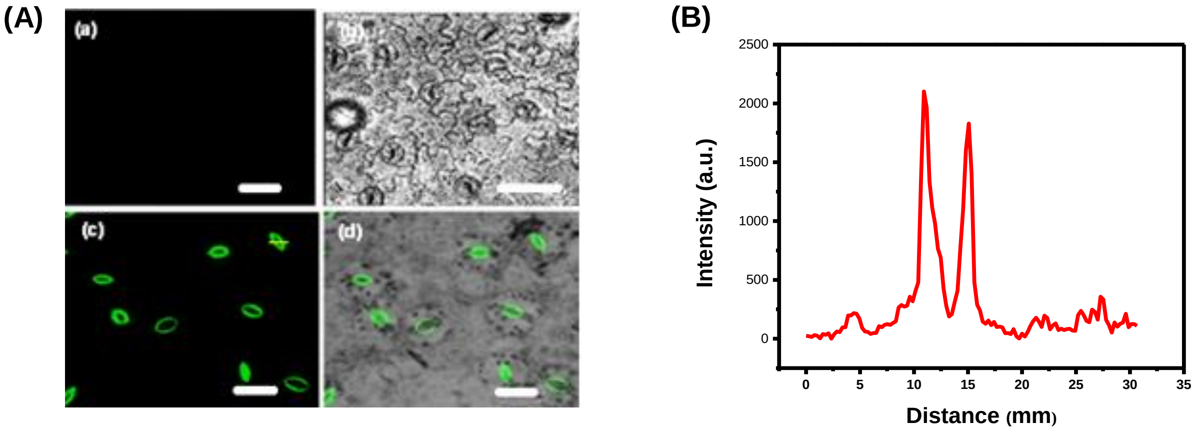

Imaging of mammalian and bacterial cells

In order to demonstrate the generality of DADQs as florescence probes for bio-imaging, we have carried out the fluorescence imaging experiments with live DU145 human prostate cell lines (live mammalian cells) and cyanobacteria (fixed cells) using BT_2 and BBEDQ respectively.

DU145 cell line

DU145 cell lines were stained by directly introducing 15 µl of the aqueous solution of the BT_2. Cell lines were immediately washed with PBS and imaged in a CLSM. The dye molecules entered the cells and stained it completely (Fig. S11).

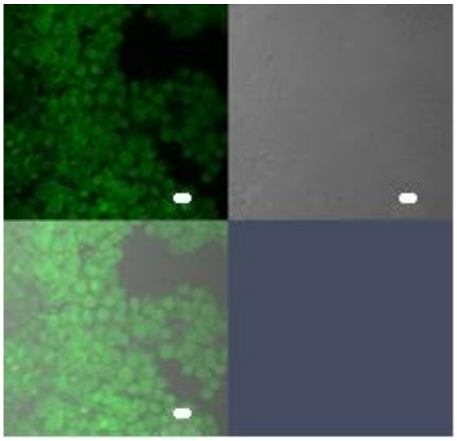

Figure S11. CLSM images (fluorescence and overlay of fluorescence with bright field) of the stained DU145 cell lines using BT_2. Scale bar: 20 µm.

Cyanobacteria cell

As BT_2 was found to be less efficient in this case, imaging was carried out using another DADQ derivative, 7,7-bis(benzylamino)-8,8-dicyanoquinodimethane (BBEDQ).[S13] 5 µl of 5 mM DMSO solution of BBEDQ was added on 0.2 ml of synechocystis pcc 6803 cells in buffer; the cells were imaged in a CLSM. The dyes entered into the cells and stained them completely.

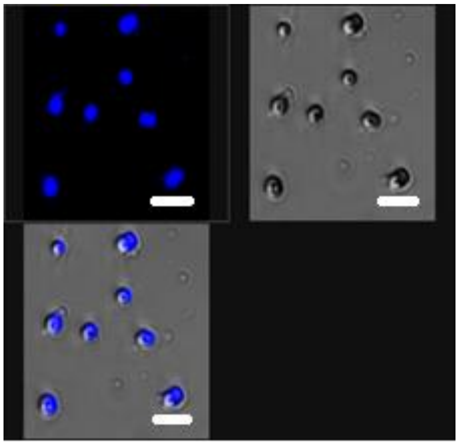

Figure S12. CLSM images (fluorescence and overlay of fluorescence with bright field, different magnification) of the stained synechocystis pcc 6803 cells by using 5 mM DMSO solution of BBEDQ. Scale bar: 5 µm.

S13. Chandaluri, C. G. & Radhakrishnan, T. P. Zwitterionic diaminodicyanoquinodimethanes with enhanced blue-green emission in the solid state. *Opt. Mater*. **34**, 119–125 (2011).

Photostability studies

The dyes were excited at 488 nm and the fluorescence monitored. BT_2 is found to be very photo-stable; DPZDQ is slightly less stable.

Figure S13. Fluorescence intensity variation of **(a)** BT_2 and **(b)** DPZDQ with irradiation time.

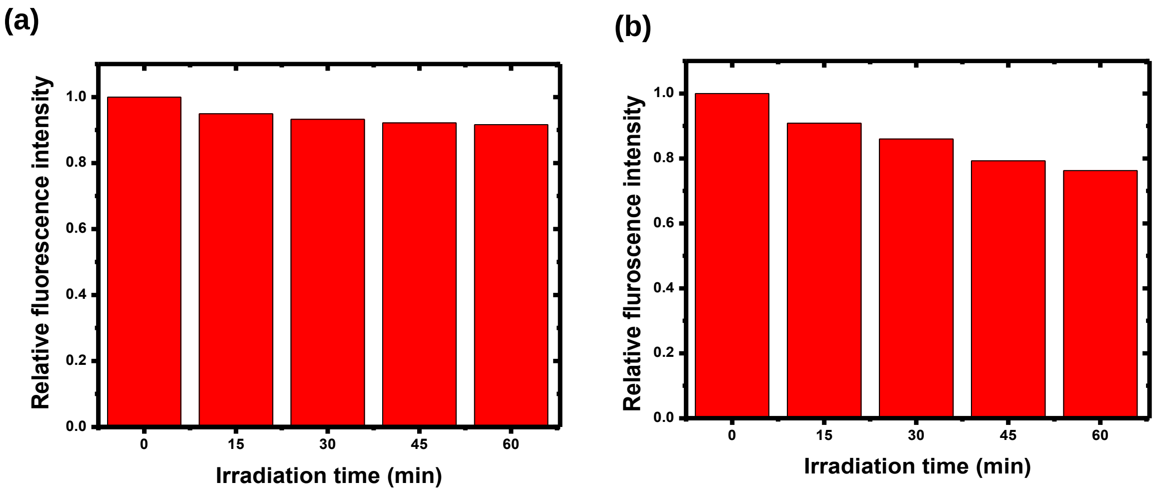

Cytotoxicity assay

Cytotoxicity assay was carried out by Pondicherry Center for Biological Sciences, Pondicherry, India (http://http://pcbscience.webs.com/). 2 - 4 × 10^5 HeLa cells were seeded in a 96-well cell culture plate with DMEM medium (Himedia) containing 1% anti-mycotic antibiotic (Himedia) and 10% FBS (Himedia), and incubated for 24 h in a CO_2 incubator at 37°C. The cells were treated initially with the test sample (BT_2, DPZDQ) in different concentrations (25, 50, 100, 250, 500 µg/mL) and incubated for 24 h. The medium was aspirated from the cells at the end of the treatment period. 0.5 mg/mL MTT and 1% PBS were added and incubated at 37˚C for 4 h. After incubation, the medium containing MTT was discarded from the cells. The crystals formed were dissolved in 100 µL of DMSO; the absorbance at 570 nm was measured using a micro-plate reader to estimate the cell viability. IC_{50} values of BT_2 and DPZDQ are found to be 1468 ± 345.4 µg/ml and 790.70 ± 98.73 µg/ml respectively

Figure S14. Percent cell viability and inhibition of L929 cell colony formation in presence of different concentrations of **(a)** BT_2 and **(b)** DPZDQ at 24 h.

(a)

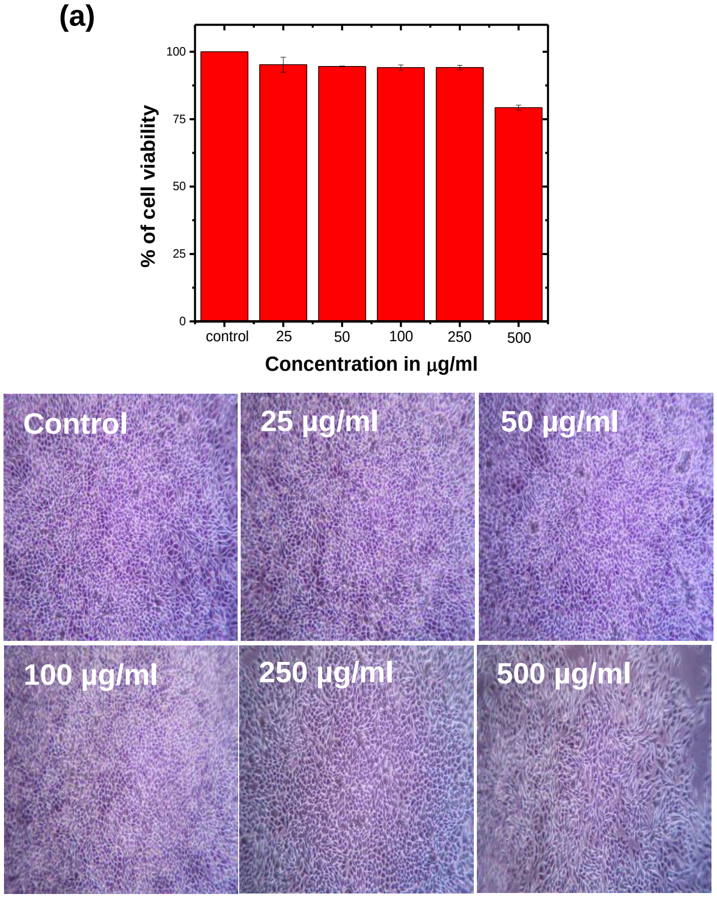

(b)

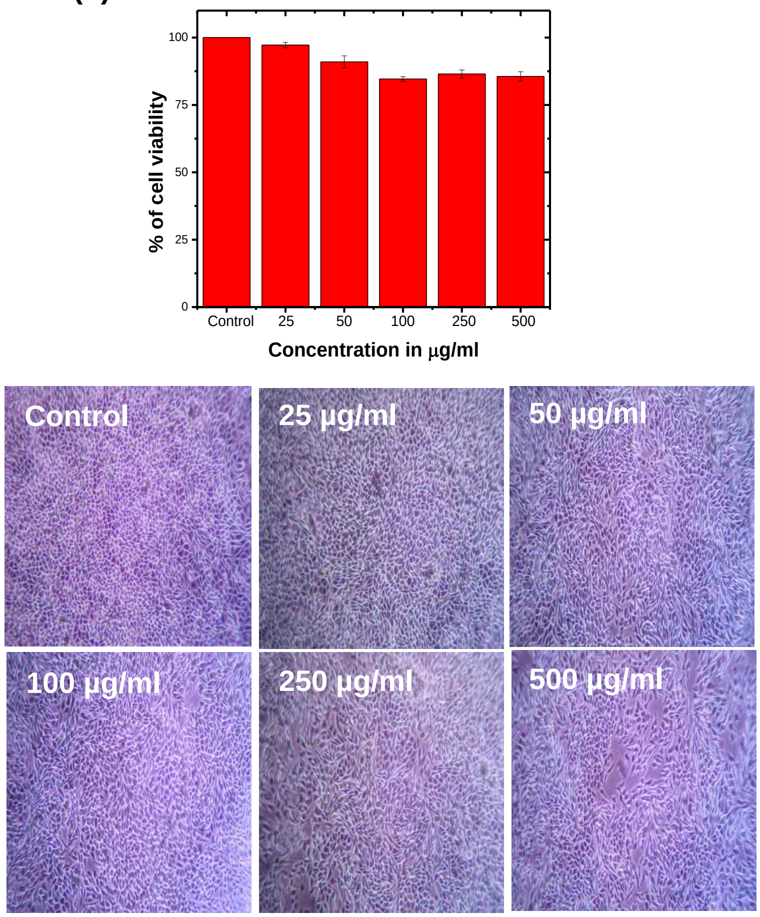
100
75
50
25
0
% of cell viability
Control
25
50
100
250
500
Concentration in μg/ml
Control
25 μg/ml
50 μg/ml
100 μg/ml
250 μg/ml
500 μg/ml

Figure S15. Percentage cell viability and inhibition of HeLa cell colony formation in presence of different concentrations of **(a)** BT_2 and **(b)** DPZDQ; the corresponding plots to determine the IC_{50} values are also shown. IC_{50} values are calculated by extra platting the curve.

(a)

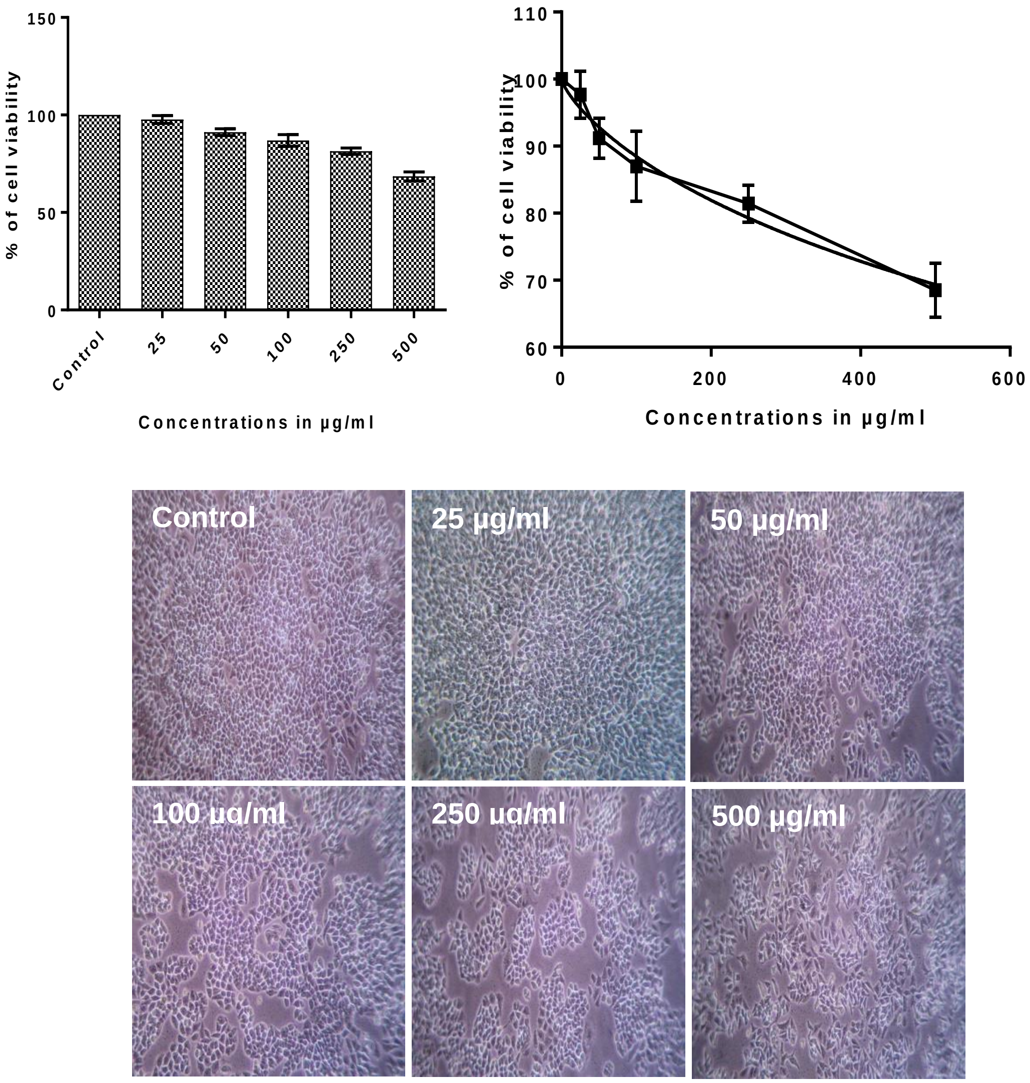

(b)

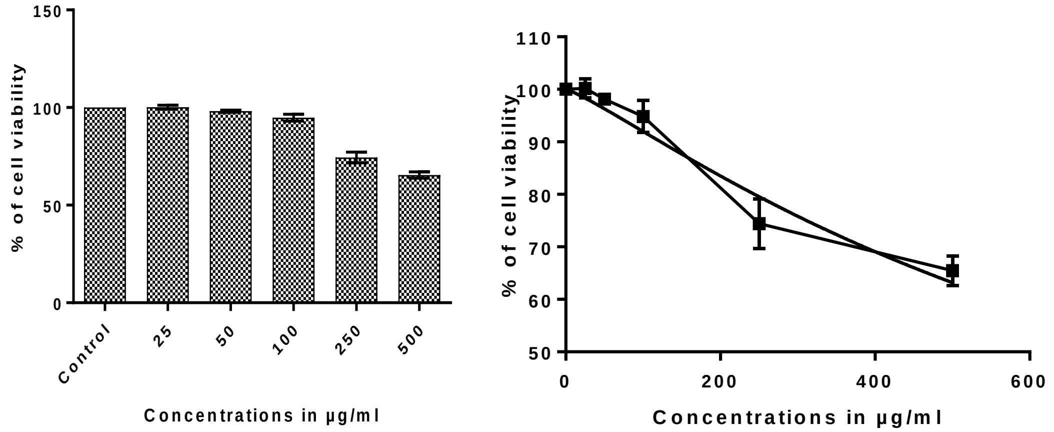
150
100
50
0
% of cell viability
Control
25
50
100
250
500
Concentrations in µg/ml
110
100
90
80
70
60
50
0
200
400
600
% of cell viability
Concentrations in µg/ml

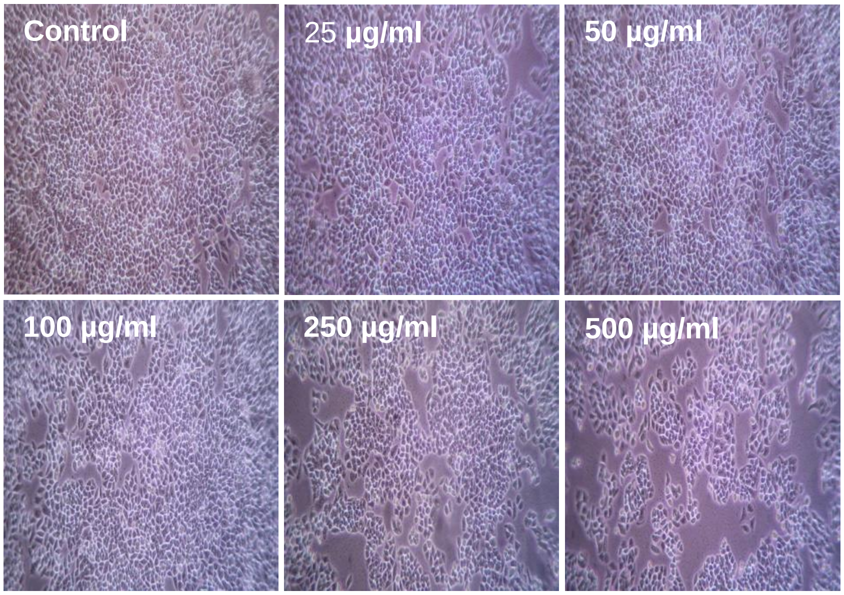
Control
25 µg/ml
50 µg/ml
100 µg/ml
250 µg/ml
500 µg/ml
